# Recurrence of vulval intraepithelial neoplasia following treatment with cidofovir or imiquimod: results from a multicentre, randomised, phase II trial (RT3VIN)

**DOI:** 10.1111/1471-0528.15124

**Published:** 2018-02-09

**Authors:** CN Hurt, SEF Jones, T‐A Madden, A Fiander, AJ Nordin, R Naik, N Powell, M Carucci, A Tristram

**Affiliations:** ^1^ Centre for Trials Research Cardiff University Cardiff UK; ^2^ School of Medicine Cardiff University Cardiff UK; ^3^ Centre for Women's Global Health Royal College of Obstetricians & Gynaecologists London UK; ^4^ East Kent Gynaecological Oncology Centre Queen Elizabeth the Queen Mother Hospital Margate UK; ^5^ Northern Gynaecological Oncology Centre Queen Elizabeth Hospital Gateshead UK; ^6^ Wellington Regional Hospital Wellington New Zealand

**Keywords:** Cidofovir, imiquimod, long‐term follow up, recurrence, vulval intraepithelial neoplasia

## Abstract

**Objective:**

To compare the recurrence rates after complete response to topical treatment with either cidofovir or imiquimod for vulval intraepithelial neoplasia (VIN) 3.

**Design:**

A prospective, open, randomised multicentre trial.

**Setting:**

32 general hospitals located in Wales and England.

**Population or Sample:**

180 patients were randomised consecutively between 21 October 2009 and 11 January 2013, 89 to cidofoovir (of whom 41 completely responded to treatment) and 91 to imiquimod (of whom 42 completely responded to treatment).

**Methods:**

After 24 weeks of treatment, complete responders were followed up at 6‐monthly intervals for 24 months. At each visit, the Common Terminology Criteria for Adverse Events (CTCAE) v3.0 was assessed and any new lesions were biopsied for histology**.**

**Main outcome measures:**

Time to histologically confirmed disease recurrence (any grade of VIN).

**Results:**

The median length of follow up was 18.4 months. At 18 months, more participants were VIN‐free in the cidofovir arm: 94% (95% CI 78.2–98.5) versus 71.6% (95% CI 52.0–84.3) [univariable hazard ratio (HR) 3.46, 95% CI 0.95–12.60, *P* = 0.059; multivariable HR 3.53, 95% CI 0.96–12.98, *P* = 0.057). The number of grade 2+ events was similar between treatment arms (imiquimod: 24/42 (57%) versus cidofovir: 27/41 (66%), *χ*2 = 0.665, *P* = 0.415), with no grade 4+.

**Conclusions:**

Long‐term data indicates a trend towards response being maintained for longer following treatment with cidofovir than with imiquimod, with similar low rates of adverse events for each drug. Adverse event rates indicated acceptable safety of both drugs

**Tweetable abstract:**

Long‐term follow up in the RT3VIN trial suggests cidofovir may maintain response for longer than imiquimod.

## Tweetable abstract

Long‐term follow up in the RT3VIN trial suggests cidofovir may maintain response for longer than imiquimod.

## Introduction

Vulval intraepithelial neoplasia (VIN) is a chronic, premalignant condition affecting the vulval skin. The age‐standardised incidence is approximately one per 100 000 women, with a peak at 30–49 years of age, and has risen over recent decades.^1^, ^2^ VIN is usually associated with high‐risk types of human papillomavirus (HPV) (>80%), most commonly HPV 16, but may also be related to lichen sclerosus.^3^ VIN can be divided into grades 1, 2 and 3, depending on the proportion of the epithelium containing undifferentiated cells, with VIN 3 displaying full thickness neoplasia.^4^ Symptoms may be severe and include pain, itching and dyspareunia, with treatment often required on these grounds alone.^5^ Rates for progression to invasive disease are difficult to estimate, as most women undergo surgery to remove the disease, but may be up to 5% per year, or 1–2% with surgery.^6^ Surgery is currently the standard treatment, but may be associated with significant morbidity^5^, ^7^ and recurrence rates are high (reported at 30–56%),^8^, ^9^ meaning that multiple surgeries are often required, which can cause significant physical and psychosexual morbidity. Alternative treatments are being sought.

The RT3VIN trial published in 2014 was a randomised phase II trial investigating the safety and efficacy of two novel topical therapies, cidofovir and imiquimod, in the treatment of VIN.^10^ Cidofovir is a nucleoside analogue with antiviral properties; imiquimod is an immune response modifying medication licensed to treat anogenital warts. The trial reported when its primary endpoint (biopsy‐proven VIN at 6 weeks post‐treatment) was mature. Between 21 October 2009 and 11 January 2013, 180 participants were enrolled to the study from 32 general hospitals located in Wales and England. At the post‐treatment assessment visit, a complete proven histological response had been achieved by 46% of patients on both cidofovir and imiquimod.

An important factor in the treatment decision‐making process is risk of recurrence, and research assessing long‐term follow up of patients treated with cidofovir for VIN 3 is lacking. Imiquimod is more extensively studied, but only a few small studies have reported follow‐up data and recurrence rates vary from 0–50%, with a follow‐up period ranging from 10 to 60 months.^11^, ^12^, ^13^, ^14^, ^15^, ^16^, ^17^, ^18^

The protocol for RT3VIN included follow up of complete responders for 2 years to assess late treatment toxicity and recurrence rates. The primary objective of this paper is to compare the recurrence rates after complete response to topical treatment.

## Methods

The trial design, treatment options, eligibility criteria and follow‐up modalities have previously been reported in detail.^10^ In summary, the trial included patients with the following key eligibility criteria: over 16 years of age; biopsy‐proven VIN 3 (including visible perianal disease not extending into the anal canal) within the last 3 months (including HPV DNA testing in the biopsy); no early invasive disease; no pregnancy; no impaired renal function; and no previous failure of imiquimod or cidofovir. Patients were randomised [1:1 minimisation with a random element (80:20) stratified by treating hospital, unifocal or multi‐focal disease, and first or recurrent disease] to receive either imiquimod or cidofovir topical treatment and to apply it three times a week for 24 weeks. Assessments during the treatment period (weeks 6, 12, 18 and 24 of treatment) included clinical assessment of lesions using adapted RECIST (see supplementary material in original paper^10^). Patients attended for their Post Treatment Assessment Visit (PTAV) 6 weeks after the end of treatment (a maximum of 30 weeks after the start of treatment) or, if earlier, 6 weeks after a complete response or disease progression (using adapted RECIST) was found. Assessments at the PTAV included a biopsy assessment of histological response. Follow‐up visits to assess recurrence rates, continued only for those participants who had a histologically complete response at the PTAV, were performed at 6, 12, 18 and 24 months post complete response. These assessments included adverse events (National Cancer Institute Common Terminology Criteria for Adverse Events (CTCAE) version 3.0), clinical examination and, if a lesion was present, a biopsy for histology (although HPV DNA testing was not done). The trial was registered (ISRCTN 34420460) and approved by a UK multicentre ethics committee and individual informed consent was obtained from all participants. A patient representative was involved in the design and management of the study. Cancer Research UK funded the trial (CRUK/06/024) and ensured external peer review for scientific quality but had no role in conducting it or writing up the results.

All statistical analyses were pre‐planned and conducted using STATA SE 14. A recurrence was defined as ‘new VIN’ of any grade, as further treatment may be administered to prevent progression to higher grades. Some lesions were not biopsied, so their VIN status was unknown (although they were noted as being either clinically suspicious or not) and some biopsies were inconclusive; thus a sensitivity analysis was conducted using ‘new lesion’ (including those not biopsied and inconclusive biopsies) as a recurrence. We calculated time to recurrence from date of the PTAV to the time when a recurrence occurred. Patients who were recurrence‐free were censored at the time they were last known to be recurrence‐free. We estimated recurrence time distributions with the Kaplan–Meier method and compared recurrence rates with hazard ratios (HR) from Cox regression in univariable models and multivariable models. In multivariable models we included the treatment effect and randomisation stratification variables (with centre as a shared frailty effect), which were the only variables thought potentially to influence recurrence *a priori*. We tested the proportional hazards assumption of each model with Cox–Snell residuals and Schoenfeld's global test.

## Results

**Figure 1:**
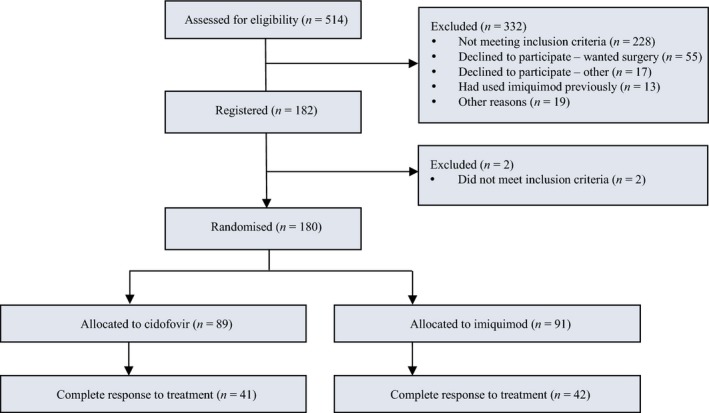
CONSORT flow diagram of trial participants.

The analysis was conducted when all complete responders (of which there were 83) had had their post‐treatment assessment visit more than 2 years previously (Figure 1). Characteristics of these patients are shown in Table 1.

**Table 1:** Patient characteristics of complete responders at baseline and post‐treatment assessment

	Cidofovir (*n* = 41)	Imiquimod (*n* = 42)
**At baseline (pretreatment)**		
Immunocompromised		
Yes	1 (2)	2 (5)
No	40 (98)	40 (95)
Smoking status		
Current	24 (59)	23 (55)
Previous	13 (32)	10 (24)
Never	4 (10)	9 (21)
Disease focality		
Unifocal	24 (59)	20 (48)
Multifocal	17 (41)	22 (52)
Sum of longest lesion diameters (mm)	35 (25–45)	37 (28–60)
Time from current diagnosis of VIN to randomisation (days)	37 (18–70)	42 (25–61)
Recurrent VIN		
Yes	19 (46)	18 (43)
No	22 (54)	24 (57)
Time from first diagnosis of VIN to randomisation (months)	66 (27–141)	85 (22–117)
Number of previous treatments (applicable to patients with recurrent disease only)		
0	0 (0)	2 (5)
1	7 (17)	7 (17)
2–4	10 (24)	9 (21)
6	1 (2)	0 (0)
Unknown	1 (2)	0 (0)
Previous other anogenital neoplasia		
Cervical intraepithelial neoplasia	12 (29)	7 (17)
Vaginal intraepithelial neoplasia	4 (10)	2 (5)
Anal intraepithelial neoplasia	4 (10)	1 (2)
None	21 (51)	31 (74)
Missing	0 (0)	1 (2)
HPV DNA‐positive		
Yes	31 (76)	32 (76)
No	6 (15)	6 (14)
Missing biopsy findings	4 (10)	4 (10)
HPV 16 DNA‐positive		
Yes	27 (66)	26 (62)
No	10 (24)	12 (29)
Missing biopsy findings	4 (10)	4 (10)
**At post‐treatment assessment**		
Age (years)	50 (45–54)	50.5 (46–57)

Data are number of patients (%) or median (IQR). VIN, vulval intraepithelial neoplasia.

### Adverse events

There were no grade 4+ adverse events during follow up (Table 2). There was no evidence of a difference between trial arms in either the proportion of complete responders experiencing any grade 2+ adverse event during follow up [imiquimod: 24/42 (57%) versus cidofovir: 27/41 (66%), *χ* ^2^ = 0.665, *P* = 0.415] or any grade 3+ during follow up [imiquimod: 3/42 (7%) versus cidofovir: 6/41 (15%), *χ* ^2^ = 1.204, *P* = 0.272].

**Table 2:** Adverse events during follow up

Expected adverse events	Cidofovir (*n* = 41)				Imiquimod (*n* = 42)			
	Grade 1–2		Grade 3		Grade 1–2		Grade 3	
	*n*	%	*n*	%	*n*	%	*n*	%
Fatigue	11	27	4	10	14	33	0	0
Pruritus	15	37	2	5	12	29	0	0
Ulceration	0	0	0	0	0	0	0	0
Pain in vulva	8	20	1	2	4	10	0	0
Headache	5	12	0	0	4	10	3	7
Muscle pain	6	15	0	0	9	21	0	0
Proteinuria	0	0	0	0	1	2	0	0
**Other adverse events** a								
Anxiety	0	0	1	2	0	0	0	0
Flu‐like symptoms	0	0	1	2	0	0	0	0

a Included if at least one patient had an event of grade 3 or higher, or if grade 1–2 adverse events in more than 10% of the population were present in any column. No grade 4 or 5 adverse events reported.

### Time to recurrence

The length of follow up was similar in each trial arm (cidofovir: median 18.2 months, 95% CI 17.5–19.0; imiquimod: median 18.8 months, 95% CI 17.9–20.4) and was a median of 18.4 months after the PTAV (95% CI 18.1–19.0 overall).

Table 3 shows the nature of the first new lesions and VIN found during follow up. No malignant lesions were found. There were no instances of VIN increasing in grade during follow up, so first VIN represents worst VIN during follow up.

**Table 3:** Nature of first new lesion and first VIN recurrence events

	Cidofovir (*n* = 41)		Imiquimod (*n* = 42)	
	*n*	%	*n*	%
No new lesions found during follow up	30	73	20	48
**First new lesion found but not biopsied**				
Not suspicious	5	12	6	14
Suspicious	1	2	2	5
Unknown	0	0	2	5
**First new lesion found and biopsied**				
VIN1	0	0	2	5
VIN2	1	2	1	2
VIN3	2	5	7	17
No VIN	1	2	1	2
Inconclusive	1	2	1	2

**Figure 2:**
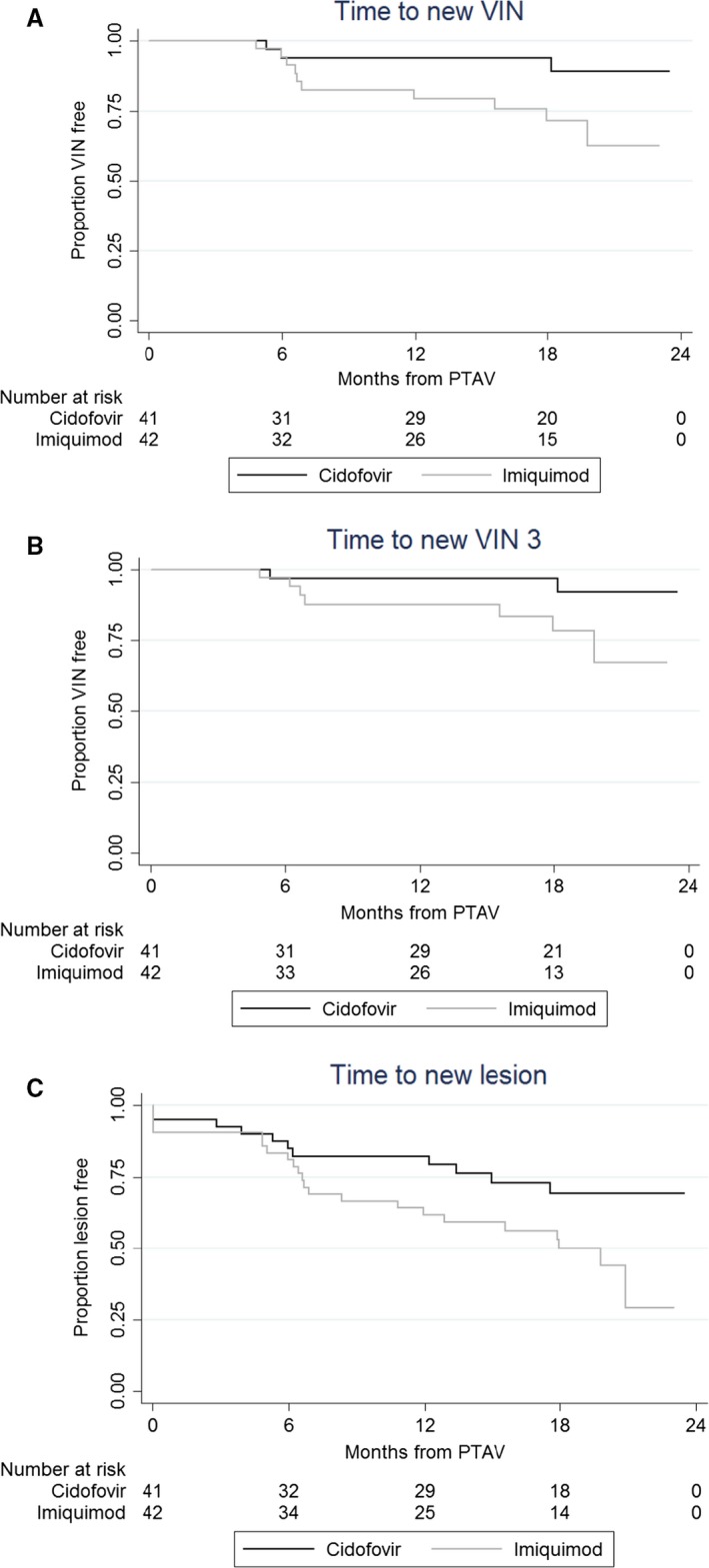
Time to recurrence by trial arm.

There was some evidence that the time to new VIN was shorter in the imiquimod arm (univariable HR 3.46, 95% CI 0.95–12.6, *P* = 0.059) (Figure 2a, Table 4). At 18 months, 71.6% of complete responders on imiquimod (95% CI 52.0–84.3) and 94.0% of complete responders on cidofovir (95% CI 78.2–98.5) remained VIN‐free. In a multivariable model, there was some evidence that imiquimod (HR 3.53, 95% CI 0.96–13.0, *P* = 0.057) and no evidence that either multifocal (HR 1.80, 95% CI 0.60–5.42, *P* = 0.294) or recurrent disease prior to treatment (HR 1.36, 95% CI 0.45–4.08, *P* = 0.584) were associated with shorter time to new VIN in complete responders. In a sensitivity analysis we also looked at time to VIN 3 recurrences only (Figure 2b, Table 4) and found a similar association with imiquimod (multivariable HR 4.72, 95% CI 0.96–23.3, *P* = 0.056). In further sensitivity analyses of time to any VIN, we also included baseline HPV DNA status (to indicate whether the original disease was differentiated versus usual VIN) and previous other anogenital neoplasia (found to be slightly imbalanced between treatment groups, as shown in Table 1). In the univariable models, neither was found to be associated with time to new VIN (HPV DNA positive: HR 0.88, 95% CI 0.19–4.05, *P* = 0.875; previous other neoplasia: HR 0.33, 95% CI 0.07–1.48, *P* = 0.147). In the multivariable model, the strength of the treatment effect was maintained (imiquimod HR 4.39, 95% CI 0.87–22.3, *P* = 0.074, *n* = 75).

**Table 4:** Univariable and multivariable Cox regression for time to recurrence (new lesion or new VIN)

Variable	Category	*n*	New lesion						New VIN						VIN3					
			Univariable			Multivariable			Univariable			Multivariable			Univariable			Multivariable		
			HR	95% CI	*P*	HR	95% CI	*P*	HR	95% CI	*P*	HR	95% CI	*P*	HR	95% CI	*P*	HR	95% CI	*P*
**Trial arm**	Cidofovir	41																		
	Imiquimod	42	2.04	0.99–4.20	0.055	1.9	0.92–3.94	0.084	3.46	0.95–12.6	0.059	3.53	0.96–13.0	0.057	3.98	0.82–19.2	0.086	4.72	0.96–23.3	0.056
**Recurrent disease prior to treatment**	No	46																		
	Yes	37	0.78	0.39–1.56	0.488	0.84	0.42–1.68	0.609	1.16	0.39–3.45	0.791	1.36	0.45–4.08	0.584	2.01	0.50–8.05	0.326	2.54	0.62–10.4	0.194
**Focality of disease prior to treatment**	Unifocal	44																		
	Multifocal	39	2.83	1.37–5.85	0.005	2.75	1.33–5.71	0.007	1.90	0.63–5.70	0.251	1.80	0.60–5.42	0.294	1.48	0.39–5.6	0.562	1.48	0.39–5.6	0.569

We also conducted a sensitivity analysis looking at time to ‘new lesion’. There was some evidence that the time to new lesion was shorter in the imiquimod arm (univariable HR 2.04, 95% CI 0.99–4.20, *P* = 0.055) (Figure 2c, Table 4). Four complete responders in the imiquimod arm and three complete responders in the cidofovir arm had new lesions present at their post‐treatment assessment visit, which explains the initial drops in the proportion of patients who were lesion‐free. At 18 months, 50% of complete responders on imiquimod (95% CI 33.6–64.5) and 69% of complete responders on cidofovir (95% CI 51.2–82.0) remained lesion‐free. In a multivariable model, there was some evidence that imiquimod (HR 1.9, 95% CI 0.92–3.94, *P* = 0.084), strong evidence that multifocal disease prior to treatment (HR 2.75, 95% CI 1.33–5.71, *P* = 0.007) and no evidence that recurrent disease prior to treatment (HR 0.84, 95% CI 0.42–1.68, *P* = 0.609) were associated with shorter time to new lesion in the complete responders.

## Discussion

### Main findings

The long‐term follow up of complete responders in RT3VIN suggests a trend towards response being maintained for longer in patients who were given cidofovir than imiquimod. There was no evidence of a difference in the rates of adverse events during the 2 years after assessment of initial response, there were no grade 4+ events, and the rates of grade 3 events were very low. At 18 months after complete response at PTAV, 71.6 and 94.0% of patients remained VIN‐free (i.e. recurrence rates of 28.4 and 6.0%) with imiquimod and cidofovir, respectively.

### Strengths and limitations

There is a lack of research investigating the long‐term outcome of patients treated with both these medications and the few studies available often present relatively small numbers or short duration of follow up. Regarding imiquimod, this study represents the largest with long‐term follow up data and the findings are in line with those reported in the literature to date. A trial of imiquimod versus placebo found that, of the 24 patients on imiquimod, nine had a complete response, eight of whom (88.8%) remained VIN‐free after 7.3 years.^16^ A non‐randomised study compared imiquimod with cold knife excision and demonstrated complete response in 46.9% (15/32) patients treated with imiquimod, of which 33.3% (5/15) had developed a recurrence by 60 months.^13^ Regarding cidofovir, the only study reporting any long‐term follow up of patients found recurrence rates of 11.1% (1/9) at 168 days; however, that study only considered low‐grade intraepithelial neoplasia.^19^

This was a phase II study and recurrence rates were a secondary endpoint. As shown in Figure 1, only complete responders were followed up, so selection bias may be occurring; other potentially prognostic variables were therefore included in multivariable analyses. Furthermore, although this research indicates that cidofovir may be superior, it is not currently available for topical administration and was formulated specifically for this clinical trial. Additionally, follow‐up time was relatively short; 5 or 10 years would provide even more useful data. Finally, although a tissue biopsy was required to confirm VIN 3 to establish eligibility for the trial at recruitment, a biopsy was not done in all cases of new lesions during follow up. Biopsies are painful and were sometimes declined in our study, hence full data on VIN status was unavailable, necessitating sensitivity analyses.

Two methods of classification of VIN exist; both are based on histologically identifiable characteristics in a tissue biopsy. The first method was established in 1986 when the International Society for the Study of Vulvar Disease (ISSVD) developed the term VIN to describe the precursor lesions of vulval squamous cell carcinoma using terminology analogous to that used for cervical disease (CIN). This system defines classic histological features to be identified and then grades the VIN based on the degree of epithelial involvement as VIN 1, 2 or 3. It was thought that the natural history of disease was progressive from VIN 1 to VIN 3 and, in some cases, to invasive cancer. Recently, use of the term VIN 1 has been discouraged based on the lack of evidence supporting the morphological continuum of VIN 1–3 synonymous with CIN.^6^ The histological changes previously identified as VIN 1 are now thought to represent the early reactive atypia associated with new human papilloma virus (HPV) infection and are, more often than not, reversible, making labelling as a pre‐malignant state inappropriate.^4^, ^20^, ^21^, ^22^ The classification was subsequently modified in 2004 by the ISSVD to recognise the two different modes of pathogenesis leading to disease; the more common usual VIN (uVIN) being HPV‐dependent and the less common differentiated VIN (dVIN), which is HPV‐independent.^23^ The subtypes are differentiated histologically. Histological features of uVIN remain the same as those used for CIN. The new terminology (uVIN, dVIN) has not been broadly adopted in the UK as yet, and many departments still use the older classifications (VIN 1, 2 and 3), which is why it was used in this study. Additionally, as the histological characteristics of dVIN are subtle and less well defined than its uVIN counterpart, this leads to an increased likelihood of intraobserver variation (Preti et al. 2000). Hence a pragmatic decision was taken to use the VIN 1, 2, 3 classification in the current study. In the RT3VIN trial, HPV DNA testing was performed on all biopsies of the original disease prior to treatment [and thus can be used as a proxy for uVIN (HPV‐positive) and dVIN (HPV‐negative)], but it was not performed on the biopsies of recurrent disease.

### Interpretation

For the purpose of comparison, the outcomes associated with surgical excision (the current standard of treatment) are more broadly studied. The largest study to date was a cohort of 405 women with VIN 2^+^ in New Zealand, in which half were followed up for at least 5 years and one quarter followed up for at least 10 years.^24^ In all, 342 of these women had initial treatment, primarily either surgical excision or laser vaporisation (11 patients are noted as having other initial treatments, including imiquimod, or unknown initial treatment). Of those who had initial treatment, 23% of patients had a second treatment (for recurrence or initial treatment failure) within 18 months. This increased to ~40% at 5 years and ~50% at 14 years. Thus the results generated by the present study indicate that the recurrence rates seen with cidofovir complete responders may be better than with surgery.

Reported recurrences following surgical treatment are often based on the presumption that 100% of the patients responded completely in the first instance. It is quite possible that these recurrences actually represent persistent disease following the excision, particularly in view of the fact that recurrences are more common in patients with positive surgical margins,^24^ but surgery probably still represents the most efficient method of management currently available. However, given the obvious benefits of a topical treatment in terms of quality of life, future work should focus on improving the initial response to medical treatment by optimising therapy. Data from the translational component of the original RT3VIN trial have demonstrated that cidofovir and imiquimod appear to be working in two biologically distinct groups (discerned according to HPV DNA methylation levels), so patients more likely to respond to one treatment or the other could potentially be identified using this as a biomarker.^25^ Alternatively, a formulation combining the two medications could be considered. Either optimisation method could improve initial response rates using a treatment modality with potentially better recurrence rates.

## Conclusion

Cidofovir may be a better topical treatment for VIN 3 compared with imiquimod in terms of maintaining complete response. This study is the largest randomised trial to have compared topical treatments of VIN and the only trial to have long‐term follow up of VIN 3 patients treated with cidofovir, and it therefore represents the best available evidence for choosing alternatives to surgery. These data, together with other results suggesting that imiquimod and cidofovir work in biologically distinct subgroups, can be used to design future trials to optimise topical treatment to allow more women potentially to avoid surgery.

### Disclosure of interests

None declared. Completed disclosure of interests form available to view online as supporting information.

### Contribution to authorship

AF, AT, CH and NP contributed substantially to the conception and design of this work. TM, AN, RN, MC, AF and AT contributed substantially to the acquisition of the data. CH analysed the data. CH and SJ were the primary authors of the manuscript and made substantial contributions to the interpretation of the data. All the authors revised the paper and approved the final version.

### Details of ethics approval

The study was approved by the Office for Research Ethics Committees Northern Ireland (ref: 08/NIR03/82) on 23 October 2008.

### Funding

The study was funded by a grant from Cancer Research UK (CRUK/06/024).
